# Motor Hysteria

**Published:** 1906-08-11

**Authors:** 


### The Hospital

A Journal of

The Medical Sciences and Hospital Administration.

Vol. XL.—No. 1,038. SATURDAY, AUGUST 11, 1906.

### MOTOR HYSTERIA.

There can be no doubt that the rapid development of the motor industry has had a marked effect upon individuals in those sections of the community who have sufficient means to enable them to bear the expense of owning a motor vehicle. Twelve months ago the services of a skilled chaffueur were at a premium; at the present moment an advertisement will bring a superabundance of applicants, most of whom have had experience in private service. Whether this change in the supply and demand is due to the circumstance that the motor mania has reached its height, and that the trend of events is for it to gradually settle down on to more restricted lines, or whether it be due to the circumstance that all who have had experience in the use of motors realise that, for economical reasons, it is desirable that the owner should act as his own chauffeur, time must determine. There is no doubt that motor riding and driving, when kept within proper limits, is an exhilarating and healthful occupation and amusement. Excessive speed is dangerous not only to the limbs and lives of those who indulge in it, but it makes the motor an insufferable nuisance.

Motor vehicles for public use in the public streets of large towns have already won for themselves much popularity from users, despite the disadvantageous conditions under which they have started. Those conditions are due to the fact that these public motor vehicles were begun in a hurry by a number of company promoters, who, for financial reasons, rushed the manufacturers and placed vehicles upon the streets which had not been properly tested, and were imperfect in many respects. In order to protect their business omnibus proprietors and others, which had heretofore had a monopoly of the street traffic, rushed into the motor business too, and their plant was little if any better than that of the new companies. The result has been that the motor omnibus, in London especially, though it has proved popular with the user, has constituted an ever-increasing nuisance to owners of houses on the routes along which it travels, and to pedestrians from the products emitted by the machines which are at times almost unbearable. The absence of excellence in the mechanism of the motor machines, due to a lack of thorough testing, and to relatively imperfect workmanship, owing to haste in production, has caused a majority of the motor omnibuses to become a nuisance from noise to the inhabitants of London and other cities, as well as a costly luxury to the owners owing to frequent breakdowns. All these evils, or most of them, might have been prevented, and must now be removed by substituting perfected machinery for that at present in use, combined with a more efficient system of police regulation. We sympathise with Lord Kilmorey as Chairman of the Charing Cross Hospital in his effort to secure an abatement of much of the nuisance created by motor omnibuses as they are at present working in the streets of the metropolis. The magistrate at Bow Street invited Lord Kilmorey to present a memorial on the subject, and it is now being signed by owners of property throughout the Strand district.

Having pointed out the evils, and their causes, which attach to public motor vehicles, we may state our conviction that they have come to stay. The public motor omnibus is infinitely preferable, from the point of view of residents and users in towns, to the tramcar and tramway. The existing nuisance caused by motor vehicles is capable of being entirely removed. The advent of the locomotive on the railway, and of the steamship, gave rise to perturbation in the public mind, and caused at least as much outcry as has been created by the motor bus. What people are accustomed to is taken as a matter of course and received with apathy and unconcern. Were this not so the fact that every year at least 140 human beings are done to death by accidents in the streets of London, whilst upwards of 9,000 suffer bodily injury from the same cause, might reasonably be expected to afford good reason for consternation, if not for panic. In fact, the public, being unconscious of the actual facts, do not disturb themselves with trifles of this description. So we find that the advent of the motor omnibus, handicapped as it has been on its first introduction to London, because of its novelty, has created a panic and an outcry altogether in excess of the justice of the case. We have no sympathy with motor hysteria manifested through excessive use of private motor vehicles, or by excessive abuse of motor omnibuses in the streets. A civilised and intelligent nation must avail itself of everything which is calculated to facilitate the business, and to secure the well-being and happiness of the people of to-day. For this reason we welcome the motor omnibus, and we have little doubt that within a few years it will have become so popular, under improved conditions, as to practically supersede horse vehicles in the streets of large cities all over the country.

